# High expression of KIFC1 is a poor prognostic biomarker and correlates with *TP53* mutation in lung cancer

**DOI:** 10.1097/MD.0000000000037286

**Published:** 2024-03-08

**Authors:** Xiuying Li, Susu Wang, Pinglang Ruan, Ousman Bajinka, Weidong Zhang

**Affiliations:** a Pulmonary and Critical Care Medicine, Hunan Provincial People’s Hospital, The First Affiliated Hospital of Hunan Normal University, Changsha, Hunan, China; b Department of Anesthesiology, Second Xiangya Hospital, Central South University, Changsha, Hunan, China; c Department of Dermatology, Second Xiangya Hospital, Central South University, Hunan Key Laboratory of Medical Epigenomics, Changsha, Hunan, China; d School of Medicine and Allied Health Science, University of The Gambia, The Gambia.

**Keywords:** Biomarker, KIFC1, lung cancer, Targeted therapy, *TP53*-mutation burden

## Abstract

The Kinesin Family Member C1 (KIFC1) is highly expressed in a variety of tumors. Since it is linked with tumorigenesis and progression, KIFC1 has emerged as a promising candidate for targeted chemotherapies. Thus, this study aims to find out the association between KIFC1 and lung cancer. The original data were assessed from The Cancer Genome Atlas and Gene Expression Omnibus databases. Compared to normal lung tissues, both mRNA and protein levels of KIFC1 were significantly increased in lung cancer tissues. The upregulation of KIFC1 was significantly correlated with sex, pathological stage, and TMN stage. Survival analysis revealed that increased KIFC1 expression was associated with poor overall survival, first-progression survival and post-progression survival in lung cancer. Based on the Gene Ontology and Kyoto Encyclopedia of Genes and Genomes analysis, we observed that KIFC1 upregulation was linked to enrichment of the cell cycle and *TP53* signaling pathway. Additionally, the overexpression of KIFC1 was positively correlated with TP53 mutations in lung cancer. Based on real-world cohort results, western blotting and RT-qPCR showed high-KIFC1 expression in lung cancer, which may be related to the malignancy of lung cancer. Finally, experiments in vitro showed that KIFC1 inhibitor could significantly inhibit the proliferation and invasion of lung cancer cells. In conclusion, KIFC1 is a poor prognostic biomarker, and patients with high-KIFC1 levels may benefit from targeted therapy.

## 1. Introduction

Lung cancer is a common malignancy that affects human health,^[1]^ and its incidence and death rate have increased rapidly in recent years in China and in the world at large.^[2]^ From these incidence, non-small cell lung cancer (NSCLC) represents approximately 85% of all lung cancer cases^[3,4]^ while lung adenocarcinoma (LUAD) accounts for more than 40% of lung cancer incidence.^[5]^ The reported 5-year survival rate for lung cancer, according to the surveillance, epidemiology, and end results program in 2011, was 15.6%,^[6]^ and the KEYNOTE-042 Study reported 5-year overall survival rates of up to 22%.^[7]^ At present, despite the substantial advancements in immunotherapy and surgical interventions, the prognosis of LUAD is poor. Hence, there is an urgent need to identify new prognostic factors and immune-related therapeutic targets to enhance the screening, diagnosis, and treatment of NSCLC.

KIFC1 encodes Kinesin Family Member C1, a motor protein belonging to the kinesin family and is the main player in various cellular processes.^[8]^ Centrosome clustering refers to the reorganization of transient multipolar spindles into pseudo-bipolar structures. This is a well-documented mechanism that enables cancer cells to evade apoptosis.^[9]^ Additionally, Kinesin Family Member C1 (KIFC1) has been associated with cisplatin resistance and poor prognosis in cancer patients.^[10]^ A recent study indicated that the increased expression of KIFC1 may promote cell proliferation in human patients^[11]^ and increases the instability of the genetic material.^[12]^ However, the precise role of KIFC1 in lung cancer remains to be elucidated especially, whether the expansion of cancer cells caused by high expression of KIFC1 is related to tumor development and mutation in LUAD.

To this end, this study aims to conduct a comprehensive analysis of KIFC1 expression in lung cancer patients using bioinformatics tools and fresh clinical tissue samples.

## 2. Materials and methods

In this study, we conducted 1 comprehensive investigation of the association between KIFC1 and lung cancer using a combination of bioinformatics analysis, in vitro validation, and examination of fresh lung cancer tissue samples. Kaplan–Meier analysis was conducted to examine the effects of KIFC1 on prognostic survival. GSEA and Kyoto Encyclopedia of Genes and Genomes enrichment analyses were conducted to examine the potential signaling pathway of KIFC1.Then, we analyzed the connection between KIFC1 mRNA level and TP53 mutation via RNA-sequencing and tumor mutation data.

### 2.1. Database

Information on database of LUAD patients were acquired from The Cancer Genome Atlas (TCGA) database. Two independent datasets (GSE75037 and GSE116959) were obtained from the Gene Expression Omnibus (GEO) database (both data were accessed on April 28, 2023). To ensure data integrity, a series of preprocessing steps was applied, including background correction, normalization, and expression calculation. Probes in the datasets were mapped to their corresponding official gene symbols using an annotation file associated with the respective platforms. LUAD samples with incomplete clinical information were excluded from further analysis. Differential gene expression analysis was conducted using the “limma” package in R software, employing stringent criteria (|log_2_(FC)| > 1, *P* < .05) to identify differentially expressed genes (DEGs) in both GEO databases for LUAD.

### 2.2. Comparison of the KIFC1 expression level

The mRNA expression of KIFC1 in lung cancer was investigated using TCGA and GEO datasets. The CPTAC Data Portal and Human Protein Atlas databases were used to analyze the expression of KIFC1 at the protein level. The expression of KIFC1 in various cancer types was analyzed using TIMER and Human Protein Atlas databases. All databases were accessed on May 12, 2023.

### 2.3. Survival and prognostic analysis

The Kaplan–Meier Plotter database, accessed on May 12, 2023 was used to estimate the correlation between KIFC1 expression and the survival rate of patients with different clinical features of lung cancer.^[13]^

### 2.4. KIFC1 differential expression analysis

The mRNA expression level of KIFC1 in patients with LUAD and health lung tissue samples was analyzed using data obtained from TCGA and GEO databases. Based on the mRNA expression levels of KIFC1 from the TCGA dataset, the samples were categorized into 2 groups: KIFC1 high- and KIFC1 low-expression group, respectively. In addition, the mRNA expression value of KIFC1 was extracted for subsequent clinical correlation analysis.

### 2.5. Sample collection

Samples were collected from the respiratory department of Hunan Provincial People’s Hospital (Table 1). Informed consent was obtained from all the patients. The experiment was approved by the Ethics Committee of Hunan Provincial People’s Hospital (LY-2023-16).

**Table 1:** Clinical information of the patients.

Patients	Gender	Age	T	N	M	Stage	Cancer type
1	Male	73	1b	0	0	IA	LUAD
2	Male	54	1c	0	0	IA	LUAD
3	Female	53	1c	1	0	IIB	LUAD
4	Female	70	2	0	0	IB	LUAD
5	Female	75	2	2	0	IIIA	LUAD
6	Male	49	2	1	-	IIB	LUSC
7	Male	60	1b	0	0	IA	LUSC

LUAD = lung adenocarcinoma.

### 2.6. Quantitative real-time PCR (qPCR)

Trizol- and enzyme-free grinding beads were added to the EP tube containing lung cancer tissue. RNA was extracted after ultrasound treatment, and reverse transcription and qPCR was performed on lung cancer tissues using a Takara qRT-PCR kit (Takara Bio, Japan).

Forward Sequence-KIFC1 Primer: CCTCACTACAGTGCCACAGACA; Reverse Sequence-KIFC1 Primer: GAACAGCAGGAACTGGCTTCTG.

Forward Sequence-GAPDH Primer: GTCTCCTCTGACTTCAACAGCG; Reverse Sequence-GAPDH Primer: ACCACCCTGTTGCTGTAGCCAA.

### 2.7. Western blot

Lung cancer cells were lysed using protease inhibitors and radioimmunoprecipitation assay cleavage buffer. Protein lysates were separated by SDS-PAGE and transferred to polyvinylidene difluoride membranes. Rabbit anti-KIFC1 antibody (1:1000 dilution, 12313S; Cell Signaling Technology) and rabbit anti-β-actin antibody (1:5000 dilution, 66009-1-Ig; Proteintech, China) were incubated overnight at 4°C, and peroxidase-conjugated goat anti-rabbit IgG (1:5000 dilution, ab6721; Abcam) was added for 2 hours at room temperature. The membranes were washed 5 times with TBST and developed using a Tanon 5200 Chemiluminescence Image Analysis System (Tanon, China).

### 2.8. Wound-healing assay and transwell invasion assay

Human lung cancer H1299 cells were cultured in RPMI 1640 medium supplemented with 10% fetal bovine serum (FBS), 100 U/mL penicillin, and 100 µg/mL streptomycin. The cell line was grown under conditions of 37°C, 100% humidity, and 5% CO_2_.

The spectrophotometric absorbance values at 450 nm for each well were determined using the TECAN F50 UV/Vis spectrophotometer (Mannedorf, Switzerland). For the transwell assay assessing cell migration and invasion capabilities in the presence or absence of the KIFC1 inhibitor AZ82 (CAS 1449578-65-7), approximately 1.5 × 10^4^ cells per well with 600 µL of complete medium containing 10% fetal bovine serum were added to the upper and lower chambers, respectively. The cells were then treated with 300 nM AZ82. After 48 hours, cells were fixed with 3.7% formaldehyde for 15 minutes and stained with crystal violet. Subsequently, nonmigratory/invasive cells were removed from the membrane, and the cells were observed and photographed. Image J software was used to quantify the distance (μm) between scratches at different time points, and the healing area (initial scratch width − final scratch width)/initial scratch width × 100% was calculated. The cells were then placed under a microscope and images were taken in 3 randomly selected fields of view.

### 2.9. Statistical analysis

Statistical analyses were performed using SPSS 22.0 statistical software (SPSS Inc, Chicago, IL) using Students *t* test. Differential genes between cancerous tissues and adjacent tissues were analyzed by paired *t* test. Data are expressed as the mean ± SD from at least 3 independent experiments. Statistical significance is defined as * *P* < .05, ** *P* < .01, *** *P* < .001, and **** *P* < .0001.

## 3. Results

### 3.1. Analysis of the expression of KIFC1 in cancers

**Figure 1.**
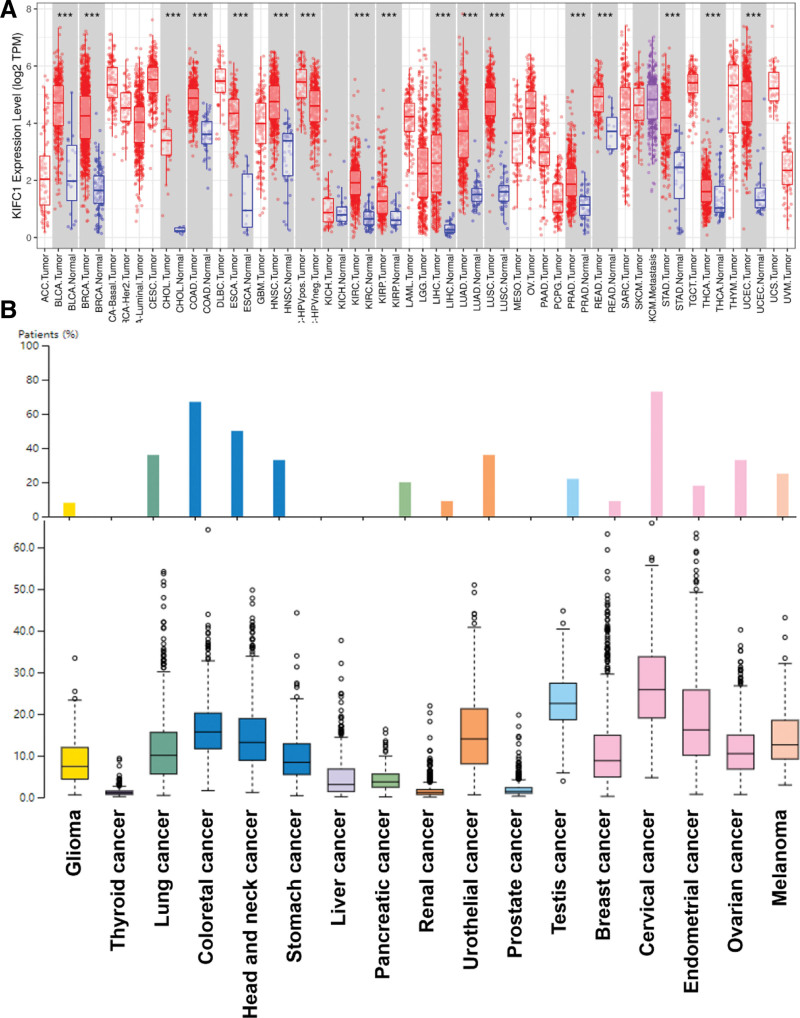
The expression of KIFC1 in various types of cancer based on (A) the TIMER database and (B) Human Protein Atlas database. *** *P* < .001. KIFC1 = Kinesin Family Member C1.

First, we analyzed the expression of KIFC1 in various types of cancer using the TIMER and Human Protein Atlas databases. The results revealed significant upregulation of KIFC1 in most cancer types, particularly melanoma, cervical cancer, ovarian cancer, endometrial cancer, lung cancer, stomach cancer, colorectal cancer, head and neck cancer, and urothelial cancer (Fig. 1A and 1B). These findings are consistent with those of previous studies that have demonstrated elevated KIFC1 expression in ovarian adenocarcinomas,^[14]^ cholangiocarcinoma,^[15]^ hepatocellular carcinoma,^[16]^ and breast cancer.^[17]^

**Figure 2.**
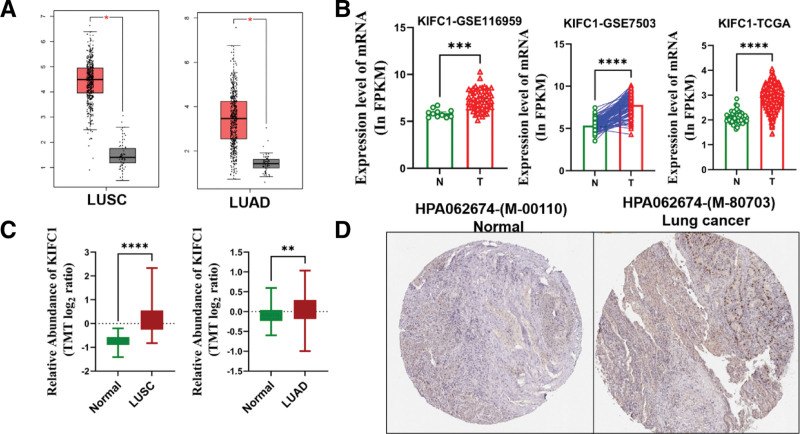
The expression of KIFC1 in lung cancer. (A) The expression of KIFC1 in LUAD and LUSC based on the GEPIA database. (B) The expression of KIFC1 in lung cancer tissues and non-tumor tissues using data from the TCGA and GEO databases (GSE116959 and GSE7503). (C) The protein expression levels of KIFC1 assessed through the CPTAC Data Portal. (D) IHC results of KIFC1 expression from the HPA database. ** *P* < .01, *** *P* < .001, and **** *P* < .0001. IHC = immunohistochemical, KIFC1 = Kinesin Family Member C1, LUAD = lung adenocarcinoma.

However, the precise role of KIFC1 in lung cancer remains to be elucidated. Therefore, we analyzed the expression of KIFC1 in lung cancer cells. Through analysis of the GEPIA database, we observed significant upregulation of KIFC1 in both LUAD and LUSC (Fig. 2A). To establish the relationship between KIFC1 and lung cancer, we evaluated the expression levels of KIFC1 in lung cancer and non-tumor tissues using data from TCGA and GEO databases (GSE116959 and GSE7503). The analysis revealed that KIFC1 expression was significantly higher in lung cancer tissues than in adjacent non-tumor tissues (GSE7503) and healthy individuals (GSE116959 and TCGA; Fig. 2B; *P* < .001). Furthermore, the protein expression levels of KIFC1, as assessed using the CPTAC Data Portal, were significantly increased in LUAD and LUSC (Fig. 2C). Consistently, immunohistochemistry (IHC) results obtained from the HPA database also demonstrated high expression of KIFC1 in lung cancer (Fig. 2D). Therefore, our analysis revealed that both the mRNA and protein expression levels of KIFC1 were significantly upregulated in lung cancer.

### 3.2. KIFC1 was the underlying diagnosis and prognosis biomarker in lung cancer

**Figure 3.**
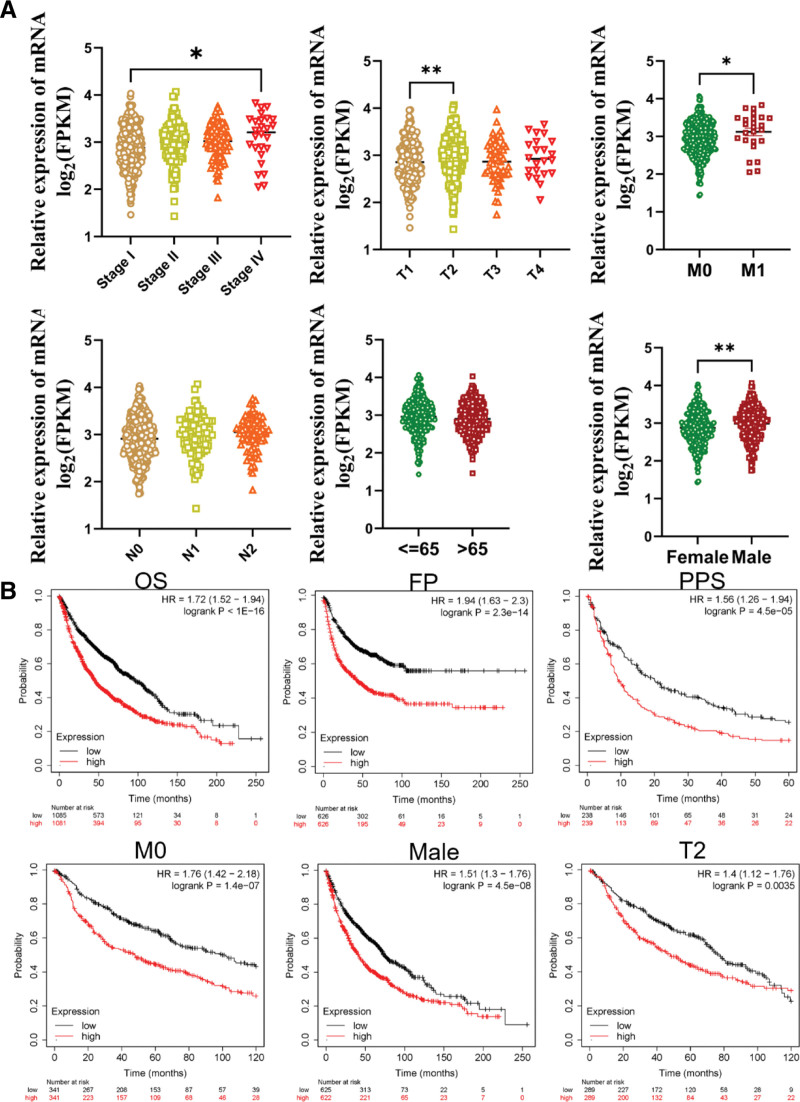
Analysis of the prognostic and diagnostic value of KIFC1 in lung cancer. (A) Correlation analysis of the relationship between KIFC1 and clinical phenotype. (B) Kaplan–Meier curve was used to analyze the prognostic value of KIFC1. * *P* < .05, ** *P* < .01. FP = first-progression survival, KIFC1 = Kinesin Family Member C1, OS = overall survival, PPS = post-progression survival.

To establish the diagnostic value of KIFC1 in lung cancer, we assessed the relationship between KIFC1 expression and the clinical phenotype (Fig. 3A). Additionally, higher expression of KIFC1 was correlated with T2 classification (*P* < .001), pathological stage IV (*P* < .05), M1 (*P* < .05), and sex (*P* < .05).

To further assess the prognostic value of KIFC1, Kaplan–Meier curves were used to compare the overall survival (OS), first-progression survival, and post-progression survival (PPS). The results showed that patients with higher KIFC1 expression had poorer prognosis (Fig. 3B). Subgroup analysis showed that high-KIFC1 expression was significantly correlated with poor prognosis in M0 (*P* < .05), male sex (*P* < .05), and T2 (*P* < .05). Thus, KIFC1 may be an underlying biomarker for the diagnosis and prognosis of LUAD.

### 3.3. Single-cell analysis of the expression of KIFC1 in different immune cells of lung cancer

**Figure 4.**
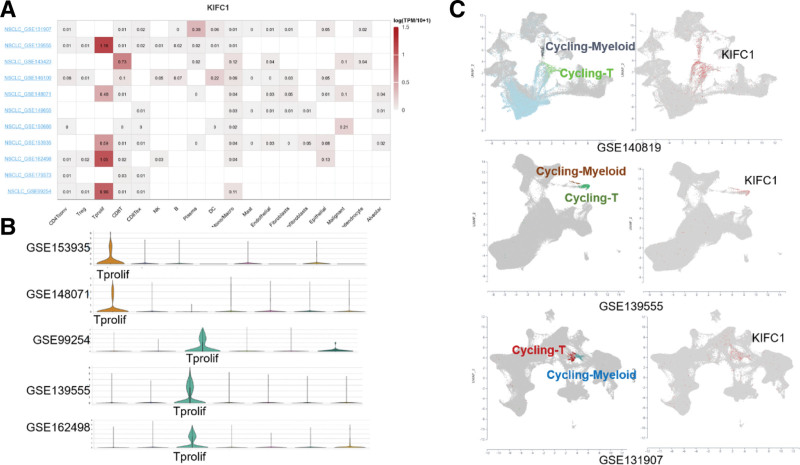
Single-cell analysis of the expression of KIFC1 in lung cancer. (A and B): The expression levels of KIFC1 from the scRNA-seq TISCH database. (C) The expression levels of KIFC1 from the scTIME Portal. KIFC1 = Kinesin Family Member C1.

Eleven independent datasets of lung cancer (NSCLC_GSE131907, NSCLC_GSE139555, NSCLC_GSE143423, NSCLC_GSE146100, NSCLC_GSE148071, NSCLC_GSE149655, NSCLC_GSE150660, NSCLC_GSE153935, NSCLC_GSE162498, NSCLC_GSE179373, NSCLC_GSE99254) from the scRNA-seq TISCH database were utilized for single-cell sequencing analysis. The aim was to investigate the association between immune cell distribution and the expression levels of KIFC1 at the single-cell level (Fig. 4A). In specific datasets, such as NSCLC_GSE139555, NSCLC_GSE148071, NSCLC_GSE153935, NSCLC_GSE162498, and NSCLC_GSE99254, high levels of KIFC1 expression were observed in T_profile_ cells (Fig. 4B). Furthermore, in the NSCLC_GSE140819, NSCLC_GSE139555, and NSCLC_GSE131907 datasets analyzed using single-cell sequencing data from the scTIME Portal, a similar trend of high-KIFC1 expression was detected in cycling T cells and cycling myeloid cells (Fig. 4C). Notably, these variations may be attributed to differences in the database annotation standards. Overall, our findings established a significant correlation between KIFC1 expression levels and immune cells, particularly proliferating T cells, thereby implying potential implications for immunotherapy.

### 3.4. The potential mechanisms of KIFC1 in LUAD

To investigate the biological function of KIFC1, we divided TCGA data into high- and low-expression groups. This was based on the expression values for 253 patients with LUAD in each group. Differential gene analysis was performed on the 2 groups to identify DEGs that were subsequently subjected to functional analysis.

**Figure 5.**
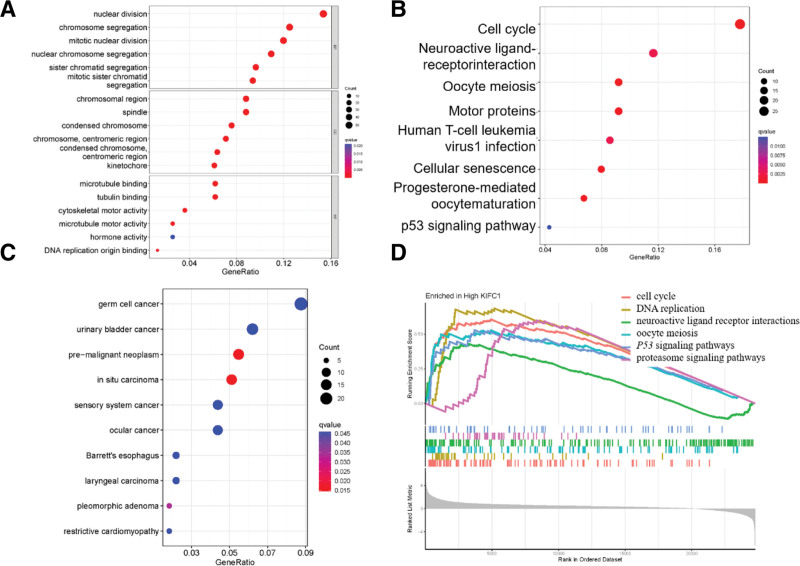
Functional enrichment analysis of KIFC1 in lung cancer, including (A) GO, (B) KEGG, (C) DO, and (D) GSEA. DO = Disease Ontology, GO = Gene Ontology, GSEA = Gene Set Enrichment Analysis, KEGG = Kyoto Encyclopedia of Genes and Genomes, KIFC1 = Kinesin Family Member C1.

Gene Ontology term annotation analysis (Fig. 5A) of the DEGs revealed that KIFC1 was associated with various biological processes such as nuclear division, chromosome segregation, mitotic nuclear division, nuclear chromosome segregation, sister chromatid segregation, and mitotic sister chromatid segregation. In terms of cellular components, KIFC1 was found to be related to chromosomal regions, spindles, condensed chromosomes, chromosome centromeric regions, and kinetochores. Moreover, KIFC1 has been implicated in molecular functions (MF) including microtubule binding, tubulin binding, cytoskeletal motor activity, microtubule motor activity, hormone activity, and DNA replication origin binding. Kyoto Encyclopedia of Genes and Genomes analysis (Fig. 5B) demonstrated the involvement of KIFC1 in various pathways, such as the cell cycle, neuroactive ligand–receptor interaction, oocyte meiosis, and motor proteins. Additionally, Disease Ontology (DO) term annotation analysis (Fig. 5C) revealed an association between KIFC1 and cancer. Furthermore, Gene Set Enrichment Analysis (GSEA) was performed by comparing the high-KIFC1 expression group with the low-KIFC1 expression group (Fig. 5D). The results indicated that high-KIFC1 expression was enriched in pathways related to the cell cycle, DNA replication, neuroactive ligand–receptor interactions, oocyte meiosis, P53 signaling, and proteasome signaling.

Overall, these findings provide insights into the potential biological functions of KIFC1, highlighting its involvement in critical cellular processes and association with cancer.

### 3.5. High expression of KIFC1 promoted *TP53* mutation in LUAD

**Figure 6.**
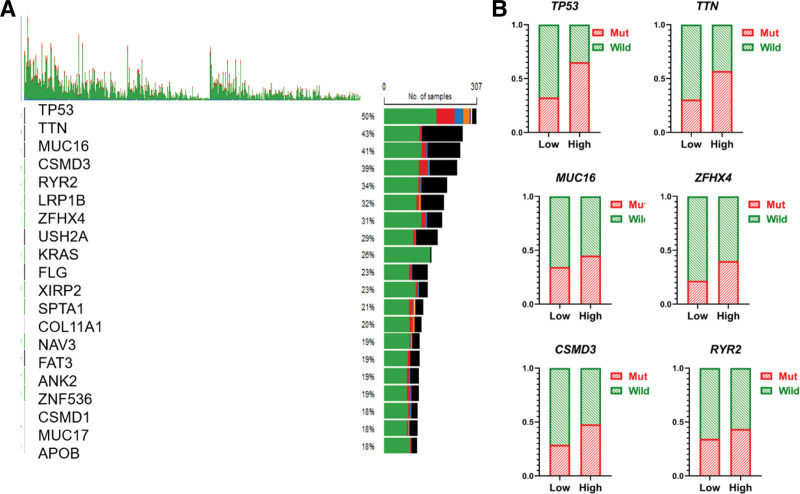
Correlation analysis between KIFC1 and key gene mutation. (A) Gene mutation in lung cancer. (B) Correlation analysis between KIFC1 and gene mutation in lung cancer. KIFC1 = Kinesin Family Member C1.

Our previous analysis showed that KIFC1 is associated with the cell cycle and *P53* signaling pathway. The tumor suppressor gene TP53 is the most commonly mutated gene in lung cancer and is associated with shorter survival.^[18]^ Therefore, we analyzed the association between KIFC1 and mutations in lung cancer cells. Our results showed that in patients with high-KIFC1 expression, the mutation rates of TP53, TTN, and other genes were significantly increased, which may be one of the mechanisms of poor prognosis in patients with high-KIFC1 expression (Fig. 6A and 6B).

### 3.6. Validation of the expression level of KIFC1 based on a real-world cohort

**Figure 7.**
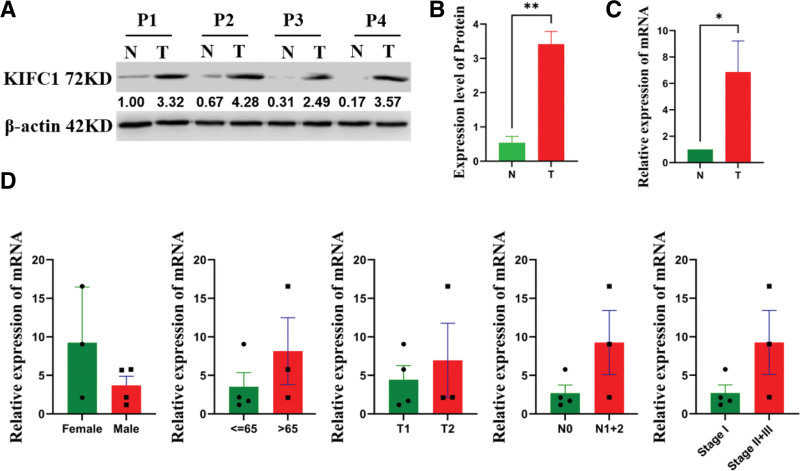
Validation of the expression level of KIFC1 based on a real-world cohort. (A and B); Western blot analysis of KIFC1 protein levels in cancerous and para-cancerous tissues. n = 4. N: para-cancerous tissues. T: cancerous tissues. (C) The mRNA expression levels of KIFC1 in cancerous and para-cancerous tissues. n = 7. N: para-cancerous tissues. T: cancerous tissues. (D) Correlation analysis of KIFC1 expression level and clinical features. * *P* < .05 and ** *P* < .01. KIFC1 = Kinesin Family Member C1.

To validate these results, we analyzed KIFC1 expression levels in 7 paired LUAD and adjacent tissues using qRT-PCR and western blotting. The protein (Fig. 7A and 7B) and mRNA (Fig. 7C) expression levels of KIFC1 in lung cancer tissues were significantly higher than those in para-carcinoma tissues, which was consistent with the results of the bioinformatics analysis. In addition, the expression level of KIFC1 was increased in advanced-stage patients, but the difference was not statistically significant, which may be limited by the sample size (Fig. 7D). These results suggest that patients with high-KIFC1 levels may benefit from targeted therapy.

**Figure 8.**
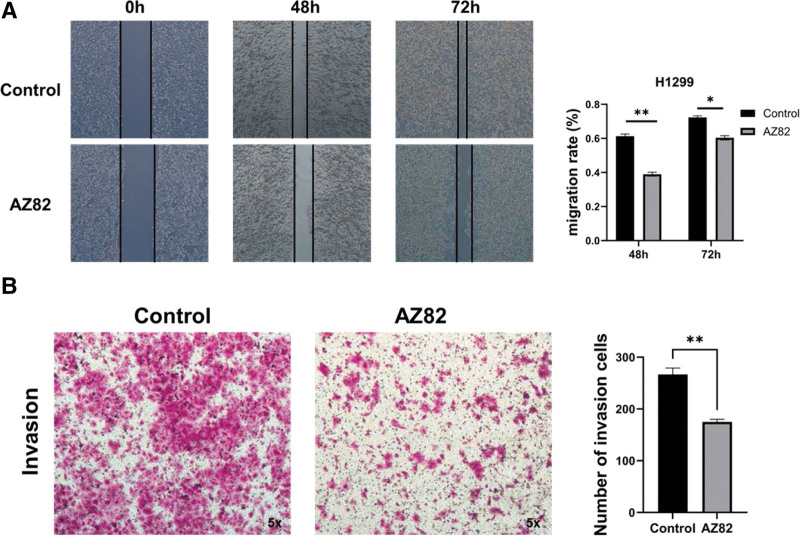
Effect of inhibitor AZ82 on the invasion and migration of H1299 cells. H1299 cells was assessed using a wound-healing assay (A) and transwell assay (B).

To delve deeper into the importance of KIFC1 in lung cancer, we conducted inhibition experiments using the KIFC1 small molecule inhibitor AZ82 (Fig. 8). Results revealed a significant inhibition of invasion in H1299 lung cancer cells with 300 nM AZ82, suggesting that the suppression of KIFC1 is advantageous in impeding the development of lung cancer.

## 4. Discussion

Lung cancer represents a significant global burden and is characterized by high incidence and mortality rates. Therefore, it is critical to enhance our scientific knowledge of lung cancer and to identify novel early diagnostic biomarkers and therapeutic targets. Bioinformatics has been an important approach to explore the role of targets in LUAD and their mechanisms.^[19]^ Among the potential targets, KIFC1, a kinesin-14 motor protein, has garnered attention due to its upregulation in multiple cancer types and its involvement in oncogenic signaling pathways.^[8,20]^ Consequently, KIFC1 has emerged as a promising candidate for targeted chemotherapies.

In this study, we conducted a comprehensive analysis of KIFC1 expression in lung cancer patients using bioinformatics tools and fresh tissue samples. Our findings demonstrated significantly elevated levels of both KIFC1 mRNA and protein in lung cancer tissues, consistent with previous reports highlighting KIFC1 overexpression in various tumor types.^[11]^ Moreover, patients with high-KIFC1 expression levels had shortened overall and disease-free survival rates. For the first time, we analyzed the correlation between KIFC1 and TP53 mutation. KIFC1 overexpression was correlated with dysregulation of the cell cycle and TP53 signaling pathway, and this was linked to increased TP53 mutation. Collectively, these results established KIFC1 as a valuable prognostic and diagnostic indicator for lung cancer.

KIFC1 plays a role in clustering additional centrosomes and in ensuring the formation of bipolar spindles during mitosis. KIFC1 activation contributes to HCC development of hepatocellular carcinoma by modulating the transcriptional activity of HMGA1. The expression of KIFC1 varies during the cell cycle and corresponds to different cell cycle phases. High-KIFC1 expression has been observed in ovarian adenocarcinomas,^[14]^ cholangiocarcinomas,^[15]^ and hepatocellular carcinomas.^[16]^ Centrosome amplification, a characteristic feature of cancer, is frequently observed in HCC patients. Their presence allows HCC cells to prevent multipolar division and promotes cell proliferation. In terms of aggressiveness, previous studies have indicated an association between KIFC1 expression and brain metastasis in primary NSCLC However, the molecular mechanisms underlying the role of KIFC1 in lung cancer remain unclear.

The genomic landscape of lung cancer reveals extensive chromosomal rearrangements and a high mutational burden, such as the functional inactivation of the tumor suppressor gene TP53.^[18]^ TP53, the most frequently mutated gene in lung cancer, leads to enhanced carcinogenic functions and is associated with shorter survival.^[21]^ Our study revealed a positive correlation between high-KIFC1 expression and TP53 mutations, suggesting a potential link between these mutations and increased KIFC1 expression. This association may be attributed to the involvement of KIFC1 in the cell cycle signaling pathways. In our study, we showed the potential of KIFC1 as 1 valuable prognostic and diagnostic marker for LUAD. We also observed associations between increased KIFC1 expression and sex, disease stage, and metastasis (TNM) stage. These findings underscore the prognostic significance of KIFC1 expression in lung cancer. Finally, we demonstrated our analysis through in vitro experiments, and the results showed that KIFC1 inhibitor could significantly inhibit the proliferation and invasion of lung cancer cells, indicating that KIFC1 had the potential as a therapeutic target.

Our findings provide compelling evidence that KIFC1 served as a novel biomarker for the malignant progression of lung cancer, which highlighted its potential relevance in LUAD progression. Although, we found that inhibition of KIFC1 inhibits cell proliferation of LUAD, we could not establish the underlying mechanisms of KIFC1 in lung cancer, which needs to be verified. Moreover, the study recommends, further study to harness the association between KIFC1 and TP53.

In conclusion, this study primarily used bioinformatics analysis and functional investigations to demonstrate that KIFC1 served as a biomarker and therapeutic target in lung cancer. These results suggested that patients with high-KIFC1 levels may benefit from targeted therapy.

## Authors contributions

XYL wrote the manuscript; PLR, OB, and SSW edited the manuscript; and WDZ revised the manuscript.

Conceptualization: Xiuying Li, Pinglang Ruan, Weidong Zhang.

Data curation: Xiuying Li.

Formal analysis: Xiuying Li, Pinglang Ruan.

Funding acquisition: Weidong Zhang.

Investigation: Weidong Zhang.

Methodology: Susu Wang, Pinglang Ruan.

Software: Xiuying Li, Pinglang Ruan.

Supervision: Weidong Zhang.

Validation: Xiuying Li, Susu Wang.

Writing—original draft: Xiuying Li.

Writing—review & editing: Ousman Bajinka, Weidong Zhang.
